# Toward a SARS-CoV-2 VLP Vaccine: HBc/G as a Carrier for SARS-CoV-2 Spike RBM and Nucleocapsid Protein-Derived Peptides

**DOI:** 10.3390/vaccines12030267

**Published:** 2024-03-04

**Authors:** Ivars Petrovskis, Dace Skrastina, Juris Jansons, Andris Dislers, Janis Bogans, Karina Spunde, Anastasija Neprjakhina, Jelena Zakova, Anna Zajakina, Irina Sominskaya

**Affiliations:** Latvian Biomedical Research and Study Centre, Ratsupites 1, LV-1067 Riga, Latvia; ivars@biomed.lu.lv (I.P.); daceskr@biomed.lu.lv (D.S.); jansons@biomed.lu.lv (J.J.); dishlers@biomed.lu.lv (A.D.); janis@biomed.lu.lv (J.B.); kspunde@biomed.lu.lv (K.S.); nastjuuuw@gmail.com (A.N.); jelena@biomed.lu.lv (J.Z.); anna@biomed.lu.lv (A.Z.)

**Keywords:** HBV genotype G, HBc, VLP, SARS-CoV-2 epitopes, immunogenicity

## Abstract

Virus-like particles (VLPs) offer an attractive possibility for the development of vaccines. Recombinant core antigen (HBc) of Hepatitis B virus (HBV) was expressed in different systems, and the *E. coli* expression system was shown to be effective for the production of HBc VLPs. Here, we used HBc of the HBV genotype G (HBc/G) as a technologically promising VLP carrier for the presentation of spike RBM and nucleocapsid protein-derived peptides of the SARS-CoV-2 Delta variant for subsequent immunological evaluations of obtained fusion proteins. The major immunodominant region (MIR) of the HBc/G protein was modified through the insertion of a receptor binding motif (RBM) from the S protein or B-cell epitope-containing peptide from the N protein. The C-terminus of the two truncated HBc/G proteins was used for the insertion of a group of five cytotoxic T lymphocyte (CTL) epitopes from the N protein. After expression in *E. coli*, the MIR-derived proteins were found to be insoluble and were recovered through step-wise solubilization with urea, followed by refolding. Despite the lack of correct VLPs, the chimeric proteins induced high levels of antibodies in BALB/c mice. These antibodies specifically recognized either eukaryotically expressed hRBD or bacterially expressed N protein (2–220) of SARS-CoV-2. CTL-epitope-containing proteins were purified as VLPs. The production of cytokines was analyzed through flow cytometry after stimulation of T-cells with target CTL peptides. Only a protein with a deleted polyarginine (PA) domain was able to induce the specific activation of T-cells. At the same time, the T-cell response against the carrier HBc/G protein was detected for both proteins. The neutralization of SARS-CoV-2 pseudotyped murine retrovirus with anti-HBc/G-RBM sera was found to be low.

## 1. Introduction

More than 773 million COVID-19 cases and 7,010,568 deaths were registered globally as of 31 December 2023, according to a WHO report [1]. Enveloped positive-sense single-strand RNA Severe Acute Respiratory Syndrome Corona Virus 2 (SARS-CoV-2), a member of the family Coronaviridae, is a causative agent of COVID-19 [2]. The dominant messenger RNA (mRNA) vaccines against SARS-CoV-2 from Pfizer-BioNTech (The Spiral, NY, USA; Mainz, Germany) and Moderna (Cambridge, MA, USA) were followed by viral vector vaccines from Janssen (Beerse, Belgium)/Johnson & Johnson (New Brunswick, NJ, USA) and AstraZeneca (Cambridge, England, UK) and by a protein subunit vaccine from Novavax (Gaithersburg, MD, USA). Despite the availability of these vaccines, there is still a need for improved, timely, adaptable, stable, and economically efficient SARS-CoV-2 vaccines.

The main target of many of the vaccines that have been designed is the spike (S) protein of SARS-CoV-2, which plays a central role in infection by interacting with angiotensin-converting enzyme 2 (ACE2) as a receptor for entry into host cells [3,4]; thus, most SARS-CoV-2 vaccines—whether they are genetic or protein vaccines—use part or all of the S protein [5].

Virus-like particles (VLPs) offer an additional perspective for COVID-19 vaccine development, as they have already been investigated for years as a promising antigen display platform [6,7]. Using recombinant VLPs as a viral-type immunogenic platform, it is possible to enhance the immune response of antigens displayed on them. VLPs have also been successfully used as adjuvants to elicit a strong immune response. By choosing appropriate types of VLPs, it is possible to stimulate both the innate and adaptive immune systems; in some cases, VLP-based vaccines without any adjuvants have been shown to stimulate humoral and cellular immunity through the MHC class I and II pathways. VLPs can be used for the presentation of B-cell and T-cell epitopes at high density, thus inducing potent humoral and cellular immune responses, respectively [8].

The most notable examples of VLP-based vaccines are the hepatitis B virus (HBV) surface antigen (HBsAg) and the human papillomavirus (HPV) vaccines. The HBV vaccine gives 80% to 100% protection to people who receive it [9,10]. The estimated effectiveness of the HPV vaccine is 83–96.1% [11,12]. Numerous studies and widespread practical applications have demonstrated that VLP vaccines are very safe. The side effects are usually minor, and they include headache, pain at the injection site, fatigue, and fever [13,14]. Currently, there is only one VLP vaccine against COVID-19—a plant-based vaccine named COVIFENZ (MT-2766)—it was developed by Medicago Inc.(Quebec City, Quebec, Canada) and was approved only in Canada. This VLP vaccine is based on the full-length S glycoprotein of strain hCoV-19/USA/CA2/2020 of SARS-CoV-2 and an AS03 adjuvant (manufactured by GlaxoSmithKline, London, England, UK) [15]. Based on trial participants aged 18 to 64, the COVIFENZ COVID-19 vaccine showed an overall efficacy of 71%, with 75% efficacy against the Delta variant and 89% efficacy against the Gamma variant of COVID-19 [16]. For comparison, the efficacy of the AstraZeneca and Pfizer vaccines after the first dose was 78% and 84%, respectively; after the second dose, the efficacy of the AstraZeneca and Pfizer vaccines was 67% and 93%, respectively [17]. Unfortunately, the production of COVIFENZ was terminated in February 2023 due to the high international market competition for COVID-19 vaccines.

According to the WHO data from 30 March 2023, 7 of 183 vaccines in the clinical stage of development and 19 of 199 vaccines in the pre-clinical stage were VLP vaccines [18]. Two of those seven candidate vaccines that were at the clinical stage used the receptor-binding domain (RBD) of the S protein as the target, while the others used the full S protein.

Recombinant virus-like particles (VLPs) of the HBV core protein (HBc) have been used for antigen display for years, as they are powerful immunogens by themselves [19,20,21]. HBc occupies a unique position among VLP carriers because of its high-level synthesis and efficient self-assembly in virtually all known homologous and heterologous expression systems, including bacteria (for a review, see references [22,23]).

For most HBV genotypes, the HBc protein is 183 aa in length, but with some exceptions—the HBc protein of HBV genotype A is 185 aa long (with the insertion of a DR between aa 153 and 154), and the HBc of HBV genotype G (HBc/G) differs due to the N-terminal DRTTLPYGLFGL extension upstream of the HBc start codon and, thus, is 195 aa long [24]. As we showed previously, the VLPs of HBc from genotypes D and G are most effectively expressed in *E. coli* in comparison with the HBc proteins of other HBV genotypes [25].

The immunogenicity, immunodominance, and especially neutralization potential of selected epitopes are crucial for the development of any effective vaccine. The RBD within the S protein of SARS-CoV-2 is responsible for most of the neutralizing antibodies against this virus [26], and the receptor-binding motif (RBM) is considered the most important region due to its direct interaction with the ACE2 receptor [27,28]. As the RBM is not glycosylated [29], it has been used in many vaccine prototypes [30,31,32,33,34]. If glycosylation does not matter, a bacterial expression system could be suitable for the production of RBM-containing fusion proteins; thus, the RBM was used for insertion into the HBc/G in our study.

Olivera et al. [35] used a bioinformatic approach to mapping B- and T-cell epitopes in the nucleocapsid protein of SARS-CoV-2 and identified a strong immunodominant B-cell epitope localized between 176 and 206 aa. In 2020, Ahlen et al. and Dutta et al. argued that the conserved nature of the N protein made it a suitable vaccine component [36,37]. The N protein was able to induce SARS-CoV- and SARS-CoV-2-specific T-cell proliferation and cytotoxic activity; thus, it was used as a representative antigen for the T-cell response in the design of a SARS-CoV-2 vaccine [38,39,40]. We reviewed recent publications related to the cytotoxic T lymphocyte (CTL) epitopes of the N protein of SARS-CoV-2, and five of them (see Section 2) were used in our study [41]. Thus, the B-cell epitope containing peptide and five CTL epitopes from the N protein were used for insertion into the HBc/G in our study.

As a vaccine platform, HBc has three insertion sites, which are the N-terminal, the C-terminal, and the major immunodominant region (MIR) (see Table 38.2. in [42]). Based on experimental investigations, the MIR, which spans amino acids 76–82, is known as the most effective region for antigen presentation. However, in each particular case, the chimeric constructs should be investigated for their VLP competence, since each particular insert has specific chemical and structural properties [43].

Our previous experience with the modification of HBc/D VLPs [44,45,46,47] convinced us to use HBc/G VLPs as epitope carriers in this investigation. Here, we present the construction, purification, and preliminary immunological characterization of HBc/G- SARS-CoV-2 fusion proteins when utilizing an *E. coli* expression system.

## 2. Materials and Methods

### 2.1. Selection of S and N Protein Fragments of SARS-CoV-2 for Insertion into the MIR of HBc/G

**Figure 1:**
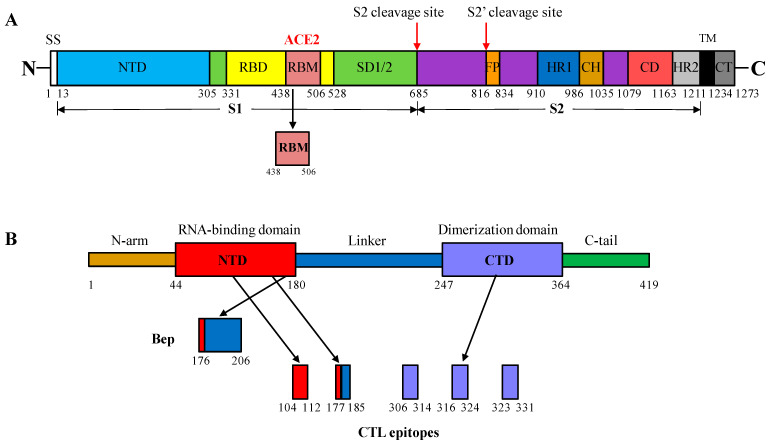
Domain organization of the SARS-CoV-2 spike (S) and nucleocapsid (N) proteins. (**A**) S protein: S1 and S2, spike protein subdomains; SS, signal peptide; NTD, N-terminal domain (light blue); RBD, receptor-binding domain (yellow); RBM, receptor-binding motif (cordovan); SD1/2, S subdomain interface (light green); FP, fusion peptide (orange); HR1, heptad repeat 1 (blue); CH, central helix (brown); CD, connector domain (pink); HR2, heptad repeat 2 (light grey); TM, transmembrane domain (black); CT, cytoplasmic tail (dark grey) (adapted from [28]). (**B**) N protein: NTD, N-terminal domain (red); CTD, C-terminal domain (lavender) and three intrinsically disordered regions: N-arm (brown), linker region (blue), and C-tail (green). The red arrows in (**A**) denote protease cleavage sites, and the black arrows in (**B**) denote peptide fragments selected from the N protein (adapted from [40]).

A. A 66-aa-long fragment representing the RBM (aa 438–506) of the S protein of the Delta variant (B.1.617.2) of SARS-CoV-2 was selected for insertion into the HBc/G MIR between aa 90 and aa 91 of the HBc/G sequence (Figure 1A).

B. The B-cell epitope-containing peptide (Bep) of the N protein representing the aa sequence SRGGSQASSRSSSRSRNSSRNSTPGSSMGTS of the Delta variant (B.1.617.2) of SARS-CoV-2 (aa 176–206) [35] was selected as another sequence of interest for insertion into the same place—between aa 90 and aa 91 of the HBc/G sequence (Figure 1B).

### 2.2. CTL Epitopes of SARS-CoV-2 Used for Insertion at the C-Terminus of the HBc/G of Different Length

Five conserved CTL epitopes from the N protein of SARS-CoV-2—LSPRWYFYY (aa 104–112), RGGSQASSR (aa 177–185), QFAPSASAF (aa 306–314), GMSRIGMEV (aa 316–324), and EVTPSGTWL (aa 323–331) [41] were selected and joined with the linker sequence AAY to design a CTL epitope string (Figure 1B).

### 2.3. Construction of Vectors for the Expression of HBc/G Fusion Proteins in E. coli

**Figure 2:**
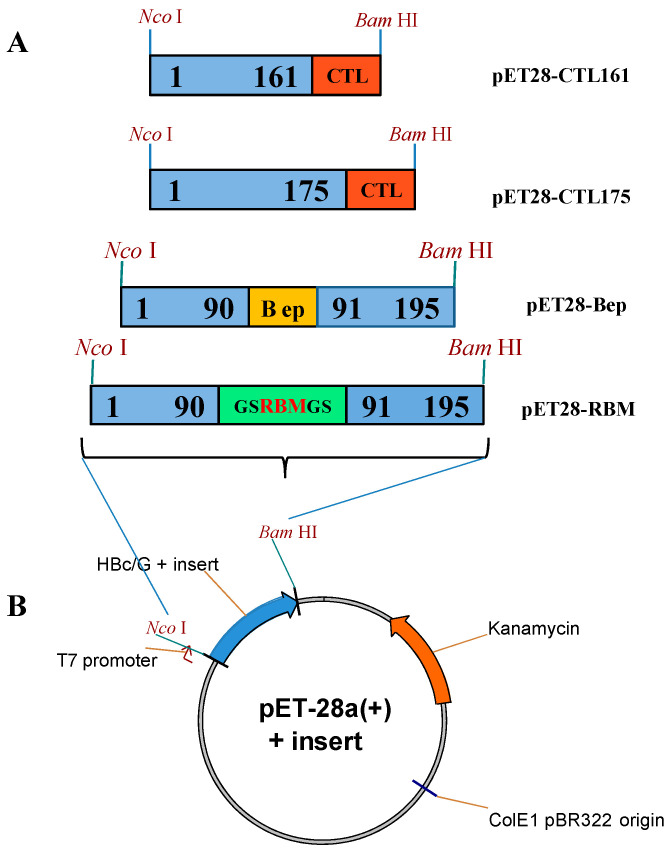
Target fusion genes and general scheme of the pET-28a(+)-based expression vector for the expression of HBc/G-SARS-CoV-2 fusion proteins. (**A**) NcoI/BamHI fragments used in the cloning (blue boxes represent the partial or full sequence of the HBc/G gene, numerals indicate the length of the HBc/G protein, the RBM fragment is shown in green, the B-cell epitope containing peptide of the N protein is shown in yellow, and the string of five CTL epitopes from the N protein is shown in red. The names of the corresponding expression plasmids are given on the right side. (**B**) General scheme of the pET-28a(+)-based expression vector. Kanamycin resistance gene is shown in orange, HBc/G is shown in blue; inserts: RBM in green, Bep in yellow, CTL string in red.

*E. coli* expression vectors based on the pET-28a(+) plasmid with the appropriate HBc/G fusion protein gene (Figure 2) were commercially obtained from BioCat GmbH (Heidelberg, Germany). The RBM sequence from the S protein and B-cell epitope-containing peptide sequence from the N protein of SARS-CoV-2 were individually inserted into a full-length codon optimized HBc/G protein as MIR insertions (plasmids pET28-RBM and pET28-Bep, respectively), and the string of five CTL epitopes from the N protein was inserted as a C-terminal extension into the two kinds of truncated HBc/G proteins: that with deleted polyarginine (PA) domain ending at aa 161 and ending at aa 175 of the HBc/G (pET28-CTL161 or pET28-CTL175, respectively, Figure 2A). A GS linker was inserted on both sides of the RBM, and AAY linker between the CTL epitopes. The target genes were expressed under the strong bacteriophage T7 promoter P_T7_ (Figure 2B). A summary of expression vectors is presented in Table 1.

### 2.4. Expression of HBc/G Fusion Proteins, Disintegration of Cells, and Solubility Test of Fusion Proteins

*E. coli* XL1 (recA1 endA1 gyrA96 thi-1 hsdR17 supE44 relA1 lac [F’ proAB lacIqZ∆M15 Tn10]) cells were used for the verification and propagation of the designed expression plasmids. Transformed cells were plated on LB agar with kanamycin (Km) at 50 µg/mL and incubated at 37 °C overnight. Five individual clones of transformed bacteria were individually transferred into tubes with 5 mL of LB medium and Km at 20 µg/mL and incubated on a shaker at 37 °C overnight, and 3 mL of culture were pelleted for plasmid extraction.

*E. coli* BL21(DE3) (F^–^ompT hsdSB (rB–, mB–) gal dcm (DE3)) was used as a host strain for the expression of the designed recombinant proteins. A verified plasmid stock was used to transform the BL21(DE3) cells, and 10 transformants were individually transferred into tubes with LB/Km at 20 µg/mL and incubated at 37 °C overnight without shaking. These individually prepared cultures served as the seed material for an expression analysis of an appropriate recombinant target protein on an analytical (5 mL of the culture in 24 mL tube) or preparative scale (200 mL of culture in a 1 L flask).

Two types of media were used for the expression analysis: (a) rich 2TY + Km 20 µg/mL medium and (b) auto-induction growth medium AIB-MAX (2TY base with added phosphates: 25 mM Na_2_HPO_4_ and 25 mM KH_2_PO_4_, 0.5% glycerol, 0.05% glucose, 0.25% lactose, Km at 20 µg/mL, and 2 mM MgSO_4_); the tubes (at a 45° angle) or flasks were placed on a shaker (200 rpm).

Cultivation in the 2TY+Km medium was initially performed at 37 °C, and 2 h after starting; IPTG was added to 1 mM, followed by cultivation at 20 °C overnight. Samples of cells for the expression analysis were taken 3 and 24 h after the addition of IPTG.

Cultivation in AIB-MAX medium was performed at 20 °C from the very beginning, and samples for analysis were taken 24 and 48 h after the start of cultivation.

For the expression analysis, normalized amounts of growing cells (two OD_540_ units) were pelleted, suspended in 200 μL of Laemmli buffer, and incubated for 10 min at 100 °C, and 10 μL of the lysate was used for SDS-PAGE (Supplementary Figure S1).

The solubility of the expressed target proteins was tested after cell disintegration with a lysozyme and urea (“soft lysis”, SL) or through ultrasound (USD). For SL, 20 OD_540_ units of cells were lysed through incubation on ice in a lysis buffer (50 mM Tris–HCl/pH 8.0, 5 mM EDTA, 0.3 mM PMSF, 1 mg/mL of lysozyme) for 30 min. After incubation, MgCl_2_ was added to the concentration of 10 mM and DNase I was added to the concentration of 20 μg/mL, followed by the addition of urea to the final concentration of 0.5 M. Treated cells were subjected to extraction by gently stirring at 4 °C for 30 min, and then the suspension was clarified through centrifugation on a benchtop centrifuge at 10,000 rpm (13,000× *g*), +4 °C for 30 min (Supplementary Figure S2A). For USD, 1 g of cells were suspended in 4 mL of HBc-lysis buffer (Tris-HCl/pH 7.2, 0.5 mM PMSF, and 0.1% Triton X100) and disintegrated with ultrasound (5 × 2 min and 10 μm), followed by the addition of 8.0 M urea to the final concentration of 0.5 M, DNase I to a concentration of 125 µg/mL, and MgCl_2_ to a concentration of 10 mM. Then, this was incubated at +4 °C for 30 min and clarified at 10,000 rpm (13,000× g) for 30 min, as described earlier [25] (Supplementary Figure S2B).

#### 2.4.1. Purification of Fusion Proteins with Insertions of the RBM or B-Cell Epitope-Containing Peptide (Bep) from the N Protein in the HBc/G MIR

After cultivation in 2TY+Km at 20 µg/mL, cells were harvested through centrifugation at 5000× *g*, +4 °C for 30 min; 3 g of cells were suspended in 30 mL of buffer (Tris-HCl/pH8, EDTA, 0.5 mM PMSF, NaCl up to 0.15 M, 0.1% Triton X100) and disintegrated via USD, as described above, followed by the addition of 8 M urea to the final concentration of 0.5 M, DNase I to 125 µg/mL, and MgCl_2_ to 10 mM; this was followed by incubation on a rotator at +4 °C for 30 min. After clarification (10,000 rpm (13,000× *g*), +4 °C for 30 min), the pellet was washed twice with 15 mL of Wash buffer (100 mM Tris-HCl, pH 8.0, 0.5 mM EDTA, 2% Triton X100, and 0.5 M urea), followed by incubation on a rotator at +4 °C for 30 min and centrifugation at 10,000 rpm (13,000× *g*), +4 °C for 30 min; after the last wash, the precipitate was subjected to stepwise extraction with urea using 15 mL of phosphate-buffered saline (PBS, 137 mM NaCl, 2.7 mM KCl, 8 mM Na_2_HPO_4_, and 2 mM KH_2_PO_4_, pH 7.4) containing increasing concentrations of urea (1.0, 2.0, and 4.0 M). Each extraction step lasted 30 min on a rotator at +4 °C, followed by centrifugation at 10,000 rpm (13,000× *g*), +4 °C for 30 min. The last extraction with 8.0 M urea was performed at RT overnight.

For the HBc/G-RBM protein, 4.0 M and 8.0 M urea extracts were combined and centrifuged at 10,000 rpm (13,000× *g*), +4 °C for 30 min, and the resulting supernatant was gradually dialyzed overnight (each step) against PBS with decreasing concentrations of urea (4.0, 2.0, 1.0, and 0.5 M). The obtained solution was centrifuged at 3000 rpm for 10 min, and the supernatant was concentrated with the use of a 30 kD Amicon filter.

In the case of the Bep MIR insertion, the 8.0 M urea extract was used for the refolding; other steps were the same as those for HBc/G-RBM protein.

#### 2.4.2. Purification of Fusion Proteins with the Insertion of the CTL Epitope String at the C-Terminus of Truncated HBc/G Proteins

After cultivation in 2TY+Km at 20 µg/mL, cells were harvested via centrifugation at 5000× *g*, +4 °C for 30 min, and 3 g of HBc/G175-CTL-protein-containing cells were disintegrated via USD and clarified as described above. Saturated ammonium sulfate solution was added to the supernatant until 35% saturation was reached, followed by incubation on a rotator at +4 °C overnight. After centrifugation at 10,000 rpm (13,000× *g*), +4 °C for 30 min, the precipitate was dissolved in 5 mL of PBS/0.5 M urea and incubated on a rotator at +4 °C for 30 min, followed by centrifugation as described above. The dissolved part was loaded on a 120 mL Sepharose 4FF/PBS column for gel-filtration (GF). Column fractions 2, 3, and 4 were pooled and applied on a 20 mL Fracto-DEAE ion-exchange (IEX) column with a chromatography buffer (PBS/0.5 M urea/0.1% Tween 20, pH 7.3).

In the case of the HBc/G161-CTL protein, after cultivation in AIB-MAX medium, 3.6 g of cells were disintegrated with USD and clarified as described above, and the obtained supernatant was directly loaded onto a 120 mL Sepharose 4FF GF column with a chromatography buffer (PBS, 0.5 M urea, 0.1% Tween 20, pH 7.3).

### 2.5. Characterization of Purified HBc/G Fusion Proteins

The purity of the preparations of fusion proteins was evaluated via SDS-PAGE after staining the 15% gels with Coomassie Brilliant Blue R-250.

The content of nucleic acids in the protein samples was evaluated via NAGE using 1% UltraPure agarose (Thermo Fisher Scientific, Waltham, MA, USA) in Tris/Borate/EDTA buffer at a pH of 8.3 (TBE, 90 mM Tris-borate, 2 mM EDTA) and staining the gel with ethidium bromide (5 µL of a 10 mg/mL EtBr stock in 100 mL of PBS).

For electron microscopy (EM), selected column fractions or VLP preparations in PBS were subjected to EM immediately or after the storage of samples at +4 °C for 1–2 days. Proteins were adsorbed onto carbon–formvar-coated copper grids and negatively stained with an aqueous solution of 1% uranyl acetate. The grids were analyzed with a JEM-1230 electron microscope (JEOL, Tokyo, Japan) at an accelerating voltage of 100 kV, and a minimum of five electrograph pictures were made per sample, magnification 100,000. Dynamic light scattering (DLS) was used to obtain particle size profiles using a Zetasizer Nano ZS instrument (Malvern Instruments Ltd., Malvern, England, UK) and the DTS software (Malvern, version 6.32). For the determination of the particle size, the ImageJ program was used.

All chemicals were obtained from Sigma-Aldrich (St. Louis, MO, USA).

### 2.6. Immunization of BALB/c Mice

Immunization of mice with purified HBc/G and HBc/G-SARS-CoV-2 proteins: HBc/G-RBM and HBc/G-Bep was performed generally as described earlier [47] using female BALB/c mice that were 8 weeks of age and obtained from the Laboratory Animal Centre, University of Tartu, Estonia, with the permission of the Latvian Animal Protection Ethics Committee (permission no. 136/2022).

Six animals in each group were subcutaneously injected with 25 µg of protein in 50 μL of PBS formulated with 50 μL of Addavax™ (InvivoGen, Toulouse, France) at days 0, 14, and 28. On day 42, the animals were bled for serum and sacrificed, and their spleens were obtained. Sera from three animals injected with only Addavax™ were used as a negative control.

### 2.7. Immunological Tests

#### 2.7.1. Enzyme-Linked Immunosorbent Assay (ELISA)

For the direct ELISA, Nunc 96-well flat-bottom plates (Thermo Fisher Scientific, Waltham, MA, USA) were coated with the appropriate proteins using 100 µL of protein solution (10 µg/mL in 50 mM sodium carbonate buffer, pH 9.6) per well followed by overnight incubation at 4 °C. Then, the plates were washed three times with a washing buffer (PBS containing 0.05% Tween-20) and blocked with 1% BSA in PBS for 1 h at 37 °C and washed again four times; 100 µL of diluted mice sera were added to the wells. Serial dilutions 1:3 were created by starting from 1:50 dilution in PBS, 0.05% Tween-20 and 0.05% BSA, and the plates were incubated at 37 °C for an additional hour. After washing four times, 100 µL/well of horseradish peroxidase-conjugated anti-mouse antibody (Sigma-Aldrich, St. Louis, MO, USA) was added at a 1:10,000 dilution. After incubation at 37 °C for 1 h, the plates were washed, and OPD substrate ((Sigma-Aldrich, St. Louis, MO, USA) was added for color development. A Multiskan Microplate Reader (Thermo Fisher Scientific, Waltham, MA, USA) was used to measure the absorbance at 492 nm.

To detect anti-RBM and anti-Bep titers, ELISA plates were coated with G-His-RBD (hRBD) and SARS-CoV-2 G-His-N protein (aa 2-220), respectively; to detect the anti-HBc/G titer, plates were coated with recombinant HBc/G VLPs. All proteins used for coating were at a concentration of 10 µg/mL in a 50 mM sodium carbonate buffer (pH 9.6, 100 µL per well). HEK-expressed G-His-RBD was described in [48] and was a generous gift from Dr. Karl D. Brune (Genie Biotech Ltd., St Clement, UK); bacterially expressed G-His-N (aa 2-220) protein was a kind in-house gift from Dr. Ilva Lieknina, BMC, Riga, Latvia. VLPs of HBc/G were obtained at the BMC, Riga, as described in [25]. The end-point titers were defined as the highest Ab dilution that resulted in an absorbance value that was three times greater than that of the negative control.

#### 2.7.2. Flow Cytometry for the Determination of SARS-CoV-2-Epitope-Specific T-Cells

For the flow cytometry (FC), splenocytes were isolated using Red Blood Cell (RBC) Lysis Buffer (Thermo Fisher Scientific, Waltham, MA, USA) and stored in liquid nitrogen using freezing media (10% DMSO, 50% FBS, and 40% RPMI culture medium). Before their use, the cells were thawed, washed, stained for viability, counted, and seeded in a U-bottom 96-well plate in a quantity of 1.5 million viable cells per well in the presence of stimulation agents: (1) a mix of SARS-CoV-2 N protein CTL epitopes representing peptides at a concentration of 2 µg/mL for each or (2) the recombinant HBc/G protein at a concentration of 10 µg/mL. The following CTL peptides—CTL epitopes of the SARS-CoV-2 N protein—were used for flow cytometry: CTL1: LSPRWYFYY; CTL2: RGGSQASSR; CTL3: QFAPSASAF; CTL4: GMSRIGMEV; CTL5: EVTPSGTWL. All peptides were purchased from Metabion (Planegg/Steinkirchen, Germany) at >85% purity and were dissolved in ultrapure water.

The mixture of mitogenic agents—phorbol myristate acetate (Sigma-Aldrich (St. Louis, MO, USA)) at 20 ng/mL and Ionomicin (Sigma-Aldrich, St. Louis, MO, USA) at 0.5 µg/mL was used as a positive control for cytokine production, and the medium alone was used as a negative control. After two hours of preincubation at +37 °C, the cell medium was supplemented with GolgyPlug reagent at a dilution of 1:1000, and the incubation was continued for another 4 h. The staining of cells for viability, T-cell surface markers (CD4, CD8), and cytokines (IFN-γ, IL-2, and TNF-α) was performed as described below and in [49]. The cytometry data were acquired using an FACSAria II cytometer (BD, Franklin Lakes, NJ, USA)with the FACSDiva™ v. 6.1.3 software (BD, Franklin Lakes, NJ, USA), exported as FCS 3.0 files, and analyzed using the FlowJo™ v. 10 software (FlowJo LLC, Ashland, OR, USA).

#### 2.7.3. Staining of Surface Markers and Intracellular Cytokines

All of the reagents for the staining of the cells were purchased from BD Biosciences (Franklin Lakes, NJ, USA). In summary, the Fc Block reagent (#553141) was introduced to the cells 10 min before the end of stimulation to prevent the non-antigen-specific binding of immunoglobulins to Fcγ receptors. Viability staining of the cells was performed using Fixable Viability Stain 660 (FSV660, cat. #564405). For the staining of the surface markers of the cells, we used FITC-conjugated anti-mouse CD8a (#553031), APC-H7-conjugated anti-mouse CD4 (#560181), and PerCP-conjugated anti-mouse CD3 (#553067) antibodies. Subsequently, the cells underwent fixation and permeabilization with the Cytofix/Cytoperm™ Fixation/Permeabilization Kit (#554714), followed by washing with Perm/Wash buffer (#554723). The cells were then stained with PE-conjugated anti-IFN-γ (#554412), BV421-conjugated anti-IL-2 (#562969), and BV510-conjugated anti-TNF-α (#563386) antibodies, which are specific to murine cytokines.

### 2.8. SARS-CoV-2-Pseudotyped Murine Retrovirus Neutralization Assay

#### 2.8.1. Plasmids and Production of SARS-CoV-2 Pseudoparticles

The technique used was based on a previously described method [50]. Both the CMV-Gag-Pol murine leukemia virus (MLV) packaging construct encoding the MLV gag and pol genes (pTG5349-Transgene) and the MLV-GFP plasmid encoding the MLV-based transfer vector with the CMV-GFP internal transcriptional unit were used as described in [50]. A pcDNA3.1-SARS2-Spike plasmid [51] encoding the full length of the SARS-CoV-2 S protein (GenBank number QHD43416.1) was used for MLV pseudotyping with the SARS-CoV-2 spike protein.

To generate the SARS-CoV-2 pseudoparticles, HEK 293-T/17 cells (ATCC, CRL-11268) were cultured in DMEM-GlutaMAX high-glucose medium supplemented with heat-inactivated fetal bovine serum (10%) and penicillin/streptomycin (50 units/mL, 50 μg/mL) in 10 cm tissue dishes (all from Gibco, Paisley, Scotland, UK). At 40% confluence, the cells were transfected with a mix of pTG5349-Transgene, CMV-GFP, and pcDNA3.1-SARS2-Spike plasmids (8 µg of each) by using the calcium phosphate precipitation method (CalPhos™ Mammalian Transfection Kit, Takara, Japan). Then, 16 h after transfection, the medium was replaced with fresh growth medium (7.5 mL/dish). The supernatant containing pseudoparticles was harvested 24 h later, filtered through a membrane with a pore size of 0.45 µm, aliquoted in 0.3–0.9 mL portions, immediately frozen in liquid nitrogen, and transferred to storage at −70 °C.

#### 2.8.2. Assays for Neutralization and Infection

Target HEK293/T17-ACE2-TMPRSS2 cells that were genetically modified to express human ACE2 and TMPRSS2 genes were obtained from the National Institute for Biological Standards and Control (NIBSC) Research Reagent Repository (UK) and cultured in IMDM medium supplemented with 2 mM glutamine, 10% heat-inactivated fetal bovine serum (Gibco, Paisley, Scotland, UK), 1 μg/mL puromycin (Promega, Madison, WI, USA), and penicillin/streptomycin (50 units/mL, 50 μg/mL) (Gibco, Paisley, Scotland, UK). To perform the infection and neutralization assay, 6 × 10^4^ cells were seeded in 0.5 mL of medium per well in 24-well plates. The next day, the infection of cells with pseudotyped MLV-SARS-CoV-2 particles and a viral neutralization assay were performed. Pooled sera of mice immunized with the HBc/G-RBM protein were analyzed at dilutions of 1:100, 1:300, and 1:900 in DMEM. Sera from all individual mice (n = 6) were also analyzed at a serum dilution of 1:300. Sera from mice immunized with HBc/G-175-CTL and HBc/G-161-CTL proteins (1:300) and naïve sera (1:100) were used as negative controls for neutralization. As a positive control of neutralization, the anti-SARS CoV-2 Spike/RBD specific polyclonal antibody (Sino Biological, Beijing, China, Cat: 40592-T62) was used at a dilution of 1:200 providing almost complete infection neutralization. MLV particles produced in the absence of the SARS-CoV-2-S-protein-encoding construct served as a negative control for the specificity of infection.

First, the dilutions of the analyzed sera samples or SARS-CoV-2-S-specific antibodies in DMEM were prepared. Next, the pseudoparticle aliquots were unfrozen at room temperature (RT), and each serum sample or antibody sample was mixed with pseudoparticles, preincubated at RT for 20 min, and added to the HEK293/T17-ACE2-TMPRSS2 cells with 250 μL per well.

After the incubation of cells at 37 °C for 5 h, 0.5 mL of complete cell culture medium was added per well, and the plate was incubated at 37 °C for a further 67 h. The transduction efficiency was determined according to the percentage of GFP-positive cells measured via flow cytometry (FACSAria II, BD, Franklin Lakes, NJ, USA) and analyzed with the FlowJo™ v. 10 software (FlowJo LLC, Ashland, OR, USA). The normalized percentage of neutralization was calculated as follows: N(y) = 100 − 100 × y/k, N(y)—neutralization (%); y represents the percentage of GFP-positive cells infected in the presence of serum or antibody; k represents the percentage of GFP-positive cells after infection with the pseudoparticle preparation alone. GraphPad Prism 8 (GraphPad Software, Inc., San Diego, CA, USA) was used for the presentation of the data, and an unpaired *t*-test was used for group comparisons.

### 2.9. Data Analysis

For the presentation of the data, GraphPad Prism 8 (GraphPad Software, Inc., San Diego, CA, USA) was used. The Mann–Whitney (nonparametric) U test was used for the statistical analysis of the immunological response, and an unpaired t-test was used for group comparisons in the neutralization assay. The *p*-value was considered statistically significant if *p* < 0.05.

## 3. Results

### 3.1. Expression of HBc/G Fusion Proteins

The results of the expression of all four designed HBc/G fusion proteins (Table 1) under the control of phage T7 late promoter P_T7_ and with the use of two different media and cultivation conditions are shown in Supplementary Figure S1A,B, respectively. For the MIR-derived HBc/G proteins, the expression levels were similar in both media after long-term cultivation, but for the C-terminally modified HBc/G proteins, the induction with IPTG showed greater expression.

The localization of the target protein in the supernatant of the lysed cells was analyzed after the soft lysis of cells with lysozyme (SL) or after cell disintegration with ultrasound (USD) (Supplementary Figure S2). After SL and USD, the main part of the proteins with MIR insertions—HBc/G-RBM and HBc/G-Bep—was found in the cell debris fraction, but with some of HBc/G-RBM being found to be soluble after USD. The HBc/G fusion proteins with the C-terminally inserted CTL string were found to be soluble after USD, and HBc/G175-CTL was mostly found in the supernatant.

### 3.2. Purification of Fusion Proteins with Insertions in the MIR of HBc/G

#### Purification and Characterization of the HBc/G-RBM and HBc/G-Bep Fusion Proteins Obtained through the Stepwise Extraction and Refolding of Insoluble Proteins

**Figure 3:**
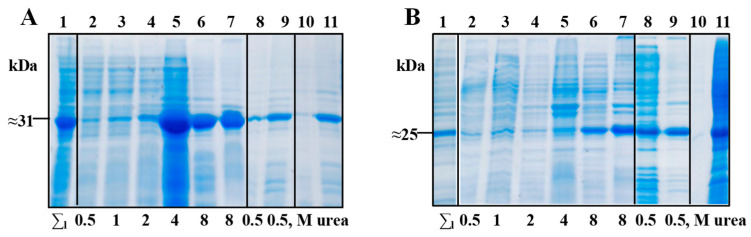
Extraction and refolding of fusion proteins with the insertion of RBM or Bep in the MIR of HBc/G. Shown is 15% SDS-PAGE for the (**A**) HBc/G-RBM protein and (**B**) HBc/G-Bep protein; staining with Coomassie Brilliant Blue G-250. 1—total cell lysate after ultrasonic disintegration (USD), (1:20); 2–6—supernatants of cell lysates after stepwise extraction of target protein with PBS and an increasing concentration of urea: 2—PBS with 0.5 M urea (1:20); 3—PBS with 1 M urea (1:5); 4—PBS with 2 M urea (1:5); 5—PBS with 4 M urea (1:1); 6—PBS with 8 M urea (1:10); 7—insoluble fraction after final extraction with PBS and 8 M urea; 8—supernatant after the final step of dialysis against PBS with 0.5 M urea (1:20); 9—remaining insoluble fraction (debris) after the final step of dialysis against PBS with 0.5 M urea (4:1); 10—the flow-through fraction after the concentration of the dialyzed protein in an Amicon 30 kD filter unit; 11—concentrated protein. Dilutions of fractions are shown in parentheses.

The stepwise extraction of the HBc/G-RBM and HBc/G-Bep proteins from the debris fraction after conducting USD on cells cultivated in the 2TY+Km medium with induction was performed with an increasing concentration of urea (PBS with 1.0, 2.0, 4.0, and 8.0 M of urea), as described in Section 2.4.1. After treatment, the protein extract was clarified via centrifugation, and the resulting supernatant was gradually dialyzed against PBS with a decreasing concentration of urea (4.0, 2.0, 1.0, and 0.5 M) to ensure the refolding of the target proteins. The results of the stepwise extraction and refolding of the HBc/G-RBM and HBc/G-Bep proteins are shown in Figure 3A,B, respectively.

The HBc/G-RBM protein after final dialysis against PBS with 0.5 M of urea was found both in soluble and insoluble fractions (Figure 3A, lines 8 and 9) whereas HBc/G-Bep protein was found mainly in the soluble fraction (Figure 3B, lines 8 and 9).

**Figure 4:**
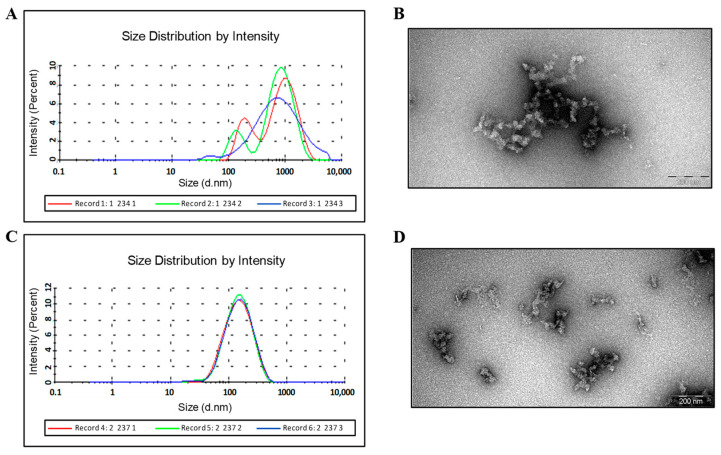
Characteristics of the purified HBc/G-RBM and HBc/G-Bep proteins according to dynamic light scattering (DLS) and negatively stained transmission electron microscopy (EM). (**A**) DLS of the HBc/G-RBM preparation (0.6 mg/mL); attenuator: 6; three independent DLS measurements are shown; the average diameter of soluble particulate material is in the range of 924–1077 nm; standard deviation 433–975 nm. (**B**) EM of the HBc/G-RBM preparation negatively stained with an aqueous solution of 1% uranyl acetate; scale bar: 200 nm, magnification: ×100,000. (**C**) DLS of the HBc/G-Bep preparation (2.0 mg/mL); attenuator: 7; three independent DLS measurements are shown; average diameter of particles: 164–170 nm; standard deviation 80–88 nm. (**D**) EM of the HBc/G-Bep preparation negatively stained with an aqueous solution of 1% uranyl acetate; scale bar: 200 nm, magnification: ×100,000.

DLS and EM data show that HBc/G-RBM and HBc/G-Bep protein preparations after refolding are still formed by aggregated particles (Figure 4B,D, respectively), which in the case of HBc/G-Bep protein, are of good homogeneity and less aggregated, as shown by DLS (Figure 4A,C, respectively).

### 3.3. Purification of HBc/G Fusion Proteins with the C-end Insertions of CTL Epitopes

#### 3.3.1. Use of Gel Filtration for the Purification of the HBc/G175-CTL and HBc/G161-CTL Proteins

Since the HBc/G175-CTL protein was found to be soluble after US disintegration (Supplementary Figure S2B), the chimeric protein was purified according to our previously established HBc-VLP purification protocol [25]. After precipitation with 35% (NH_4_)_2_SO_4_, the precipitate was solubilized, the obtained material was loaded on a Sepharose 4FF gel filtration (GF) column (Supplementary Figure S3A–C), and the combined peak fractions were further purified via ion exchange (IEX) chromatography on a Fracto-DEAE column (Supplementary Figure S4).

**Figure 5:**
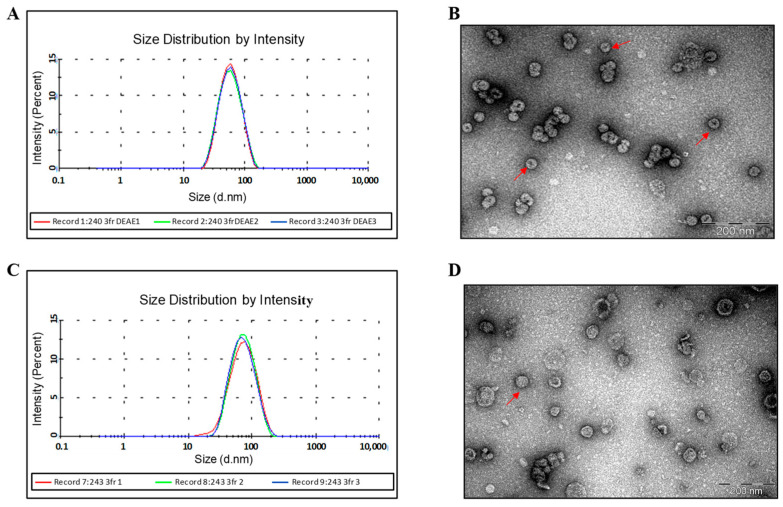
Characteristics of the purified HBc/G175-CTL and HBc/G161-CTL proteins according to dynamic light scattering (DLS) and negatively stained transmission electron microscopy (EM). (**A**,**B**) Preparation of the HBc/G175-CTL protein obtained via GF and IEX chromatography. (**A**) DLS of combined Fracto-DEAE column fractions 3 and 4; three independent DLS measurements are shown, attenuator: 7; average particle diameter 61–62 nm. (**B**) EM of the same material negatively stained with an aqueous solution of 1% uranyl acetate; scale bar: 200 nm, magnification: ×100,000. (**C**,**D**). Preparation of the HBc/G161-CTL protein after GF as a single chromatography step. (**C**) DLS of the GF column fraction 3; attenuator: 6; three independent DLS measurements are shown, average particle diameter: 77–79 nm. (**D**) EM of the same material negatively stained with an aqueous solution of 1% uranyl acetate; scale bar: 200 nm, magnification: ×100,000. VLPs are indicated with red arrows.

As for the other C-terminally modified HBc/G protein, HBc/G161-CTL, the presence of the target protein after USD was only partly found in the supernatant, but after precipitation with ammonium sulfate, this protein was resistant to solubilization (not shown). For this reason, the supernatant after USD was directly loaded on a Sepharose 4FF GF column (Supplementary Figure S4A–C). GF fraction analysis revealed that the first two fractions contained aggregates of the target protein, with the VLP-like structures found in the next two fractions (Figure 5D). Notably, the capsid material collected from these two fractions showed the presence of nucleic acids at a very low level (Supplementary Figure S4). Therefore, IEX chromatography as the following step was not considered applicable for the HBc/G161-CTL protein. GF fractions 3 and 4 were combined, and the protein was concentrated to 0.6 mg/mL using an Amicon 30 kD filtration unit. DLS and EM of this material revealed that the HBc/G161-CTL protein was in the form of capsid-like structures of high homogeneity, as shown for GF fraction 3 (Figure 5C,D).

#### 3.3.2. IEX Chromatography of HBc/G175-CTL

The combined GF fractions (2–4) of the HBc/G175-CTL protein were further purified via IEX chromatography on a Fracto-DEAE column (Supplementary Figure S5A). As shown by NAGE, VLPs were found in column fractions 3–7 (Supplementary Figure S5C), and these fractions were combined and concentrated with the use of an Amicon filter unit to a concentration of 1.5 mg/mL. EM showed the presence of capsid-like structures in the preparation (Figure 5B).

DLS of combined Fracto-DEAE fractions 3 and 4 revealed a respectable particle homogeneity in the HBc/G175-CTL preparation (Figure 5A), but the diameter of the particles was approximately two times greater than that of the wt HBc/G VLPs, with a size of around 61–62 nm. EM showed that the capsid-like structures in the preparation tended to stick together (Figure 5B).

### 3.4. Immunological Results

#### 3.4.1. B-Cell Response

**Figure 6:**
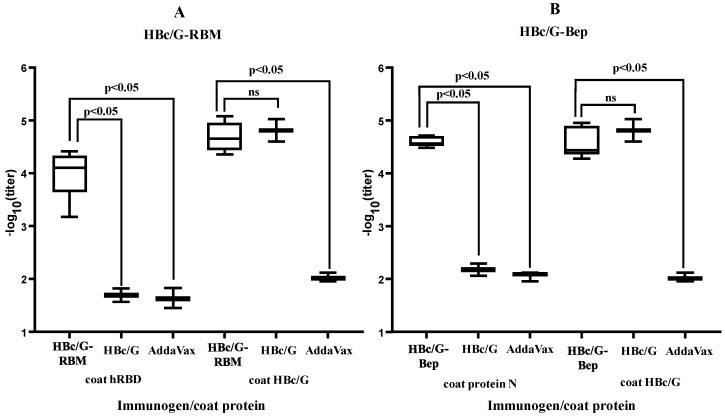
B-cell response in BALB/c mice immunized with fusion proteins with the insertion of RBM or Bep in the MIR of HBc/G. (**A**) RBD-specific and HBc/G-specific ELISA (OD_492_) titers (log_10_) for the group of mice (n = 6) immunized with HBc/G-RBM and control group of mice (n = 3) immunized with Addavax on day 42 after the first immunization. (**B**) N protein-specific and HBc/G-specific ELISA titers for the group of mice (n = 6) immunized with HBc/G-Bep and control group of mice (n = 3) immunized with Addavax on day 42 after the first immunization. For the presentation of the data, GraphPad Prism was used to create a box-and-whisker plot, and the median is shown as a line inside the box. A Mann–Whitney U test of the antibody response was performed for the statistical analysis, and *p*-values are indicated on the top. ns = not significant.

The purified fusion proteins were used for the immunization of BALB/c mice in the presence of a squalene-based oil-in-water AddaVax™ (InvivoGen, Toulouse, France) nano-emulsion as an adjuvant. The results of immunization are presented in Figure 6.

For the specific anti-SARS-CoV-2 response of the HBc/G-RBM and HBc/G-Bep proteins, the average titers of anti-RBM and anti-Bep were found to be high: 1:13,000 and 1:40,000, respectively. Concerning the response against the carrier and anti-HBc for fusion proteins with the MIR inserts, the average titers for the HBc/G-RBM and HBc/G-Bep proteins were 1:57,000 and 1:44,000, respectively, whereas in the control group of mice immunized with pure HBc/G, the average anti-HBc titer was 1:105,000.

#### 3.4.2. CTL Response

**Figure 7:**
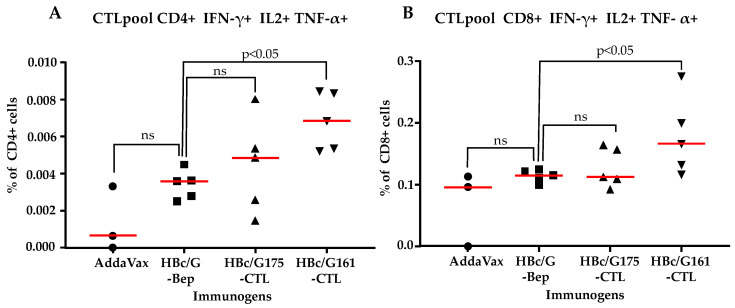
Population of CD4+ and CD8+ T-cells expressing the three cytokines IFN-γ, IL-2, and TNF-α after immunization with the HBc/G175-CTL, HBc/G161-CTL and HBc/G-Bep fusion proteins (n = 6), and control group of mice (n = 3) immunized with Addavax. Spleens of mice from each group were collected at necropsy at day 42 after the first immunization, splenocytes were isolated and subjected to the stimulation with the mix of five individual peptides of SARS-CoV-2 N protein CTL epitopes (CTL1 + CTL2 + CTL3 + CTL4 + CTL5) used in the study as a string for C-terminal insertion of HBc/G). (**A**) Production of IFN-γ, IL-2, and TNF-α by CD4+ T-cells. (**B**) Production of IFN-γ, IL-2, and TNF-α by CD8+ T-cells. GraphPad Prism was used for the presentation of the data, and the Mann–Whitney U test was used for statistical analysis; the *p*-values are indicated on the top; ns = not significant.

The production of cytokines IFN-γ, IL-2, and TNF-α in the splenocytes from immunized mice stimulated with specific antigens was analyzed via flow cytometry (Figure 7 and Supplementary Figures S6–S8).

Since the analysis of single cytokines produced by T-cells did not reveal a statistically significant response to stimulation with the mixed SARS-CoV-2 CTL peptides (Supplementary Figure S6), we used a test of the three cytokines in combination for the population of cells expressing IFN-γ, IL-2, and TNF-α. For the CD4+ and CD8+ cells expressing the three cytokines, a significant difference between the group of mice immunized with the HBc/G161-CTL fusion protein and the control group was detected (Figure 7). The difference was much more pronounced for CD8+ cells, giving indirect evidence that a specific CTL response was developed in the case of immunization with the HBc/G161-CTL protein.

An analysis of the T-cell response against the carrier when the stimulation of the splenocytes of immunized animals was performed with an unmodified recombinant HBc/G protein, specific stimulation was detected for CD4+ and CD8+ cells expressing only the TNF-α cytokine (Supplementary Figure S7).

The data that were obtained showed that only the HBc/G161-CTL protein but not the HBc/G175-CTL protein was able to induce the activation of T-cells specific to the CTL epitopes of the SARS-CoV-2 N protein in mice. However, the T-cell response against the carrier HBc/G protein was also detected in the immunizations with both the HBc/G175-CTL and HBc/G161-CTL fusion proteins.

#### 3.4.3. The Ability of Mice Sera to Neutralize SARS-CoV-2-Pseudotyped MLV Particles

**Figure 8:**
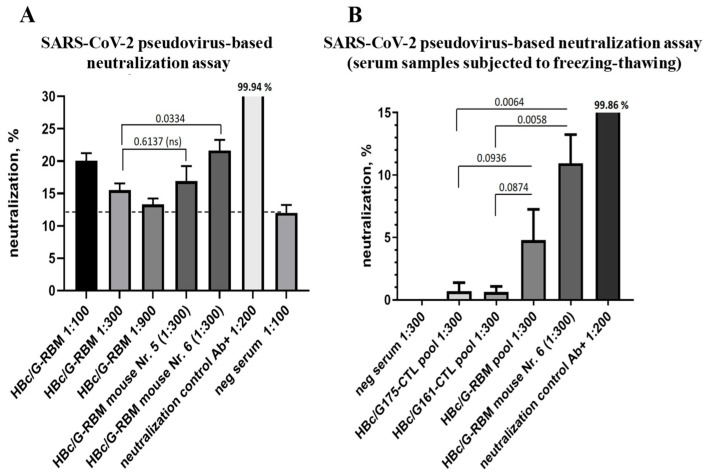
Neutralization of the pseudotyped SARS-CoV-2/MLV-GFP virus with sera from mice immunized with the HBc/G-RBM protein. Infection inhibition was tested using HEK293/T17-ACE2-TMPRSS2 cells, highly susceptible to SARS-CoV infection. (**A**) From left to right: neutralization with pooled serum samples from the groups of mice (n = 6), neutralization with the sera of individual mice that showed the highest antibody titers in ELISA (mouse Nr. 5 and mouse Nr. 6), positive neutralization control, negative serum control (serum from a naïve mouse); the dilutions of the sera are shown (1:100, 1:300, and 1:900, respectively). (**B**) Neutralization with serum samples subjected to freezing–thawing, from left to right: negative serum, the pool of sera from the mice immunized with the HBc/G-175-CTL protein, the pool of sera from the mice immunized with the HBc/G-161-CTL protein, the pool of HBc/G-RBM sera, individual serum of mouse 6, and positive neutralization control; the dilutions of the sera are shown. Cells expressing GFP as a reporter of infection were quantified via flow cytometry, and neutralization was calculated as the proportion of infected cells with respect to the control (infection without any sera). Commercially available SARS-CoV-2-neutralizing polyclonal antibodies Ab+ (Cat. No. 40592-T62, Sino Biological, Beijing, China) at a dilution of 1:200 served as a positive control for neutralization. GraphPad Prism was used for the presentation of the data and an unpaired t-test was used for group comparisons; the *p*-values are indicated.

The neutralizing activity of immunized mice sera was analyzed using SARS-CoV-2-pseudotyped MLV (murine leukemia virus) in HEK293/T17-ACE2 -TMPRSS2 cells that were genetically modified to stably produce human ACE2 receptor and TMPRSS2 transmembrane protease. The HEK293/T17-ACE2-TMPRSS2 cells were highly susceptible to SARS-CoV-2 infection [10.21769/BioProtoc.4194]. As a positive control for neutralization, the anti-SARS-CoV-2 Spike/RBD-specific polyclonal antibody (Sino Biological, Beijing, China), which provided complete neutralization at a dilution of 1:200, was used. For the neutralization test, 1:100, 1:300, and 1:900 dilutions of serum samples in DMEM were prepared. Next, the aliquots of pseudoparticles were defrosted at room temperature (RT), and 8 µL of each serum sample, antibody sample, or DMEM was mixed with 0.8 mL of pseudoparticles and preincubated at RT for 20 min, followed by the replacement of the medium of HEK293/T17-ACE2-TMPRSS2 cells with 250 µL of pseudoparticle preincubation mix per well; each sample was added in triplicate. As shown in Figure 8A, the pooled sera from the immunized mice showed relatively weak but statistically significant inhibition of the infection at a dilution of 1:100, with 20.07% inhibition for the sera of immunized mice versus 12.01% inhibition for the sera of naïve mice (negative serum showing the background of the reaction, *p* = 0.0083). At further dilutions of the pooled sera (1:300 and 1:900), the inhibition was insignificant: 15.5 ± 1.75% and 13.33 ± 0.72%, respectively. The individual mouse sera representing the highest ELISA antibody titers in the HBc/G-RBM-immunized mouse group (samples from mice 5 and 6) were also evaluated in the neutralization assay at a dilution of 1:300. As a result, increased neutralization was observed for sample 6, which reached an inhibition value of 21.66 ± 2.79%. After the freezing–thawing of the serum samples, the neutralization ability of sample 6 was lower, as it only reached a value of 10.94% (at 1:300), but this was statistically significant in comparison with the neutralization with sera from mice immunized with the HBc/G175-CTL or HBc/G161-CTL proteins, which both served as a control serum, that did not carry the RBM, with neutralization values of 0.69% and 0.62%, respectively (Figure 8B). Furthermore, the subsequent analysis of neutralization for all individual mouse sera (1:300) revealed the statistically significant pseudovirus neutralization only in the case of mouse 4 and mouse 6, which demonstrated 15,47 % (*p* = 0.0215) and 11,86 % (*p* = 0.0496) of infection decrease, respectively (Supplementary Figure S9A). Interestingly, the efficiency of neutralization did not correlate with the anti-RBD titer for each of individual mouse serum (Pearson coefficient r = −0.5847, *p* = 0.2229) (Supplementary Figure S9B). In summary, the pooled HBc/G-RBM samples equally mixed from all mouse sera in a group did not show neutralization ability comparing to HBc/G175-CTL (*p* = 0.0936) and HBc/G161-CTL (*p* = 0.0874) as controls (Figure 8B). It must be concluded that although some neutralizing effects were observed with fresh sera of mice immunized with HBc/G-RBM (Figure 8A), after freezing-thawing only two individual mice sera from this group of mice (n = 6) showed the infection inhibition in subsequent experiments. As a result, the inhibition of pseudovirus infection cannot be claimed in our study and further improvement in the vaccine candidate should be considered.

## 4. Discussion

This study aimed to use virus-like particles (VLPs) of the recombinant core antigen of HBV genotype G (HBc/G) produced in *E. coli* of SARS-CoV-2 immunogenic sequences, i.e., selected fragments from the S and N proteins of the SARS-CoV-2 Delta variant. For the presentation of foreign sequences, HBc/G was chosen from other HBc protein candidates from other HBV genotypes, since it appeared to be a technologically promising VLP candidate in terms of HBc protein production level and VLP stability in *E. coli* [25].

Two sites of HBc/G were used for the insertion—the MIR of full-length HBc/G for the insertion of the RBM of the SARS-CoV-2 S protein or the B-epitope containing peptide from the N protein and the C-end of truncated HBc/G—of the combined sequence of five CTL epitopes of the N protein. The RBM was inserted along with the most commonly used GS linkers on both sides of the RBM sequence. The flexible GS linker has been shown to improve folding and stability in several fusion protein cases [52]. String of CTL epitopes was inserted along with an AAY linker between five individual CTL epitopes. It was predicted that the AAY linkers could significantly improve the expression of certain target proteins and enhance the immunogenicity of a multi-epitope influenza vaccine designed in silico for the production in *E. coli* [53]. However, the sequence to be inserted may affect the solubility and VLP-forming ability of chimeric HBc VLPs [54]. Previously, we successfully obtained chimeric HBc VLPs using HBc/D proteins of different lengths as carriers of the HBV preS1 domain, which was inserted into the MIR of HBc [47]. When the HBc protein was used as a VLP carrier for the insertion of other viral antigens, the shortened version of HBc—the HBc149 designed with the His-tag at the N-terminus—was usually used [55,56,57]; in all described cases using the HBc149, chimeric proteins were expressed as inclusion bodies that were recovered through the use of denaturant urea with subsequent dialysis. Similarly to these reports, we faced the same problem for both of our HBc/G fusion proteins that carried the RBM or the B-cell epitope containing peptide of SARS-CoV-2 in the MIR of the full-length HBc/G protein—our proteins were also insoluble. Hence, additional steps, including denaturation with an increasing concentration of urea and refolding processes through step-wise dialysis, were necessary to obtain purified materials suitable for immunization. As for the preparation of HBc/G-Bep, a fusion protein was obtained in the form of homogenous non-VLP particles, as revealed by DLS. Soluble VLPs were found only for our HBc/G fusion proteins that carried a CTL epitope string at the C-terminus of the truncated HBc/G proteins HBc/G 161 and HBc/G175.

Until now, only two studies have been published on genetically engineered HBc-protein-based SARS-CoV-2 vaccines produced in *E. coli* [58,59]. Sazegary et al. [58] used the short RLNEVAKNL epitope of the SARS-CoV-2 S protein with G4SG4 linkers on both sides of the epitope for insertion into the MIR of a truncated, HBc149 protein. This chimeric protein was again found to make an inclusion body in the host cells and was extracted with urea in a way similar to that used in our study. However, to be used in affinity chromatography, this recombinant fusion protein was appropriately designed with a His-tag at the N-terminus of HBc149. Hassebroek et al. [59] made a more complex construct on the basis of the His-HBc149 protein: they inserted three epitopes of SARS-CoV-2: a B-cell epitope (562-FQQFGRDIADTTDAVRDPQT-581) between the N-terminal 6-His-tag and the first aa of the HBc sequence; the dual B- and T-cell epitope (371-SASFSTFKCYGVSPTKLNDL-390) in the MIR between aa 79 and aa 80; and the T-cell epitope (440-NLDSKVGGNYNYLYRLFRKSN-460) at the C-terminus of HBc149. Insoluble protein was solubilized with urea, which was followed by anion exchange chromatography and affinity chromatography. A different VLP construct based on the RNA-phage AP205 coat protein (CP) was used by Liu et al. [30], and the SARS-CoV-2 RBM domain was added C-terminally to a dimer of covalently closed AP205 CP monomers (AP205-RBM); however, this fusion protein was also found to be insoluble.

As mentioned above, the fusion proteins with the CTL epitope string at the C-terminus of the shortened HBc/G were found to be partly soluble in our study, and the use of gel filtration (GF) allowed the separation of the VLPs of the fusion protein from the protein aggregates. At the same time, the presence of nucleic acids in the VLPs of HBc/G175-CTL allowed us to use ion exchange chromatography as the next step of purification of HBc/G175-CTL protein. DLS analysis of the preparation of the HBc/G175-CTL protein revealed the presence of a homogenous material formed by VLPs of an enlarged size (61–62 nm), while the unmodified HBc/G VLPs were around 38 nm in size [25]. EM confirmed the VLP status of the purified HBc/G175-CTL fusion protein. Another C-terminally modified HBc/G protein with the CTL epitope string, the HBc/G161-CTL protein with deleted polyarginine (PA) domain, was less soluble, and it did not sustain precipitation with ammonium sulfate; the precipitate was insoluble and, thus, was only partially purified through one-step direct GF chromatography of disintegrated by US cells. It is of interest to note that the VLPs of the HBc/G161-CTL protein in the GF fractions following the aggregate fraction contained nucleic acids at a very low level confirming the absence of PA domain.

In the immunization of BALB/c mice, we used a squalene-based oil-in-water Addavax adjuvant, which was shown to be advantageous in the immunization of mice with RBD-conjugated nanoparticles [60]. At present, more than 10 different adjuvants, such as MF59, CpG, AS01, AS03, AS04, liposomes, etc have been used in human vac-cines [61]. AddaVax, which is similar to MF59, has been approved as a seasonal influenza vaccine component in Europe for people aged 65 and older [62]. AddaVax has been widely used in experimental vaccines to enhance antibody titers [63,64].

Although no exact VLPs were obtained in our study for fusion proteins carrying MIR insertions, the B-cell responses—anti-hRBD for the HBc/G-RBM protein and anti-N (aa 2-220) for the HBc/G-Bep protein in BALB/c mice—were found to be remarkably high.

Flow cytometry is the only platform that can provide multi-parametric analysis of the immune responses necessary for the most sensitive quantification of immunogenicity, the identification of functional correlates, and adequate comparison of different regimens [65]. Polyfunctional cells are characterized by their ability to secrete multiple cytokines simultaneously. This feature is believed to be crucial in shaping specific and targeted immune responses, allowing for a more diverse and adaptive defense against pathogens and diseases [66,67]. T-cell polyfunctionality has been identified in both the CD4+ and CD8+ subsets, with the cells being capable of secreting combinations of cytokines. The presence of polyfunctional T-cells of both the CD4+ and CD8+ subsets was linked with the protective effect of anti-SARS-CoV-2 vaccines on humans [68,69], through the production of interferon-gamma (IFN-γ), tumor necrosis factor-alpha (TNF-α), and interleukin-2 (IL-2) [70]. Since the analysis of single cytokines produced by the T-cells of mice immunized with our HBc/G161-CTL and HBc/G175-CTL proteins did not reveal a statistically significant response after stimulation with the mixed SARS-CoV-2 CTL peptides, we performed a combined test for the population of cells expressing three cytokines—IFN-γ, IL-2, and TNF-α—when stimulated by a pooled sample of the five CTL peptides used in this study. We found that for the CD4+ and CD8+ cells that expressed all three cytokines (IFN-γ/IL-2/TNF-α), there was a significant difference between the animals immunized with the HBc/G161-CTL fusion protein and the control group. Although it was not possible to determine the contribution of each individual peptide to the immune response in this analysis, we propose that the specific activation of polyfunctional T-cells found for the HBc/G161-CTL protein is indicative of the protective potential of this particular recombinant construct.

An important goal of any vaccine is the induction of neutralizing antibodies that can inhibit infection [33]. In our study, we tested the neutralization of SARS-CoV-2-pseudotyped MLV particles with the sera from mice immunized with the HBc/G-RBM protein. Despite the relatively high total anti-hRBD titer, the viral neutralization capacity did not reach a level that could be considered protective, since the threshold of 50% neutralization was not achieved (Figure 8). Furthermore, the analysis of individual mouse sera did not reveal a positive correlation between neutralization effects and anti-hRBM titers (Figure S9B). It is important to note that in the infection model used by Hassebroek et al. [59], the inhibition of SARS-CoV-2 infection was not achieved, despite the finding that their VLP vaccine elicited epitope-specific humoral and cell-mediated immune responses. The results of our study clearly demonstrate that highly specific antibody titers alone do not predict sufficient protection. Furthermore, the insertion of the RBM sequence into the MIR of HBc/G can lead to conformational changes in the RBM sequence, thereby potentially influencing its ability to induce effective neutralizing antibodies. However, the high immunogenicity of our recombinant RBM-containing protein has the potential to induce specific antibodies with low but specific neutralizing activity in the system of HEK293/T17-ACE2-TMPRSS2 cells expressing the human ACE2 receptor.

Bai [71] suggested that the presence of epitopes from the SARS-CoV-2 N antigen in SARS-CoV-2-spike-related vaccine designs has the potential to increase immunogenicity in next-generation SARS-CoV-2 vaccines. Our previous experience with the construction and immunological evaluation of multivalent HBc/D VLPs carrying HBV and HCV epitopes in the same HBc VLP module [72] allows us to plan the combination of B- and T-cell epitopes from SARS-CoV-2 in one recombinant HBc protein molecule.

Conclusions. This study is a novel empirical investigation of the design of full-length HBc/G-antigen-based recombinant proteins carrying SARS-CoV-2 epitopes, their production in *E. coli*, and their immunological characterization. On this stage of research we used the pseudotyped MLV/SARS-CoV-2 system to test the virus neutralization efficiency of anti-RBD sera obtained by the immunization of BALB/c mice with our HBc/G-RBM construct. The virus challenge of animals such as K18-hACE2 mice is considered the next appropriate validation step of most perspective vaccine candidates selected by pseudotyped MLV/SARS-CoV-2 system in future research.

## Figures and Tables

**Table 1:** Vectors for the expression of HBc/G fusion proteins.

Expression Vector	Fusion Protein (aa)	Inserted Sequence (aa)	Insertion Site in HBc/G
pET28- RBM	HBc/G-RBM (271 aa)	RBM, 438–506 aa region from the S protein (72 aa)	Between 90 and 91 aa (MIR)
pET28- Bep	HBc/G-Bep (226 aa)	Peptide, 176–206 aa region from the N protein (31 aa)	Between 90 and 91 aa (MIR)
pET28- CTL161	HBc/G161-CTL (232 aa)	String of five CTL epitopes from the N protein (57 aa)	After 161 aa
pET28- CTL175	HBc/G175-CTL (218 aa)	String of five CTL epitopes from the N protein (57 aa)	After 175 aa

## Data Availability

The data presented in this study are contained within the article.

## Supplementary Materials

The following supporting information can be downloaded at: https://www.mdpi.com/article/10.3390/vaccines12030267/s1. Figure S1. Expression of the designed HBc/G fusion proteins in transformed *E. coli* BL21(DE3) cells cultivated in two different expression media. Two OD_540_ units of growing cells were pelleted, suspended in 200 μL of Laemmli buffer, incubated for 10 min at 100 °C, and 10 μL of the lysate was used for SDS-PAGE analysis. (A) Cultivation of a selected transformant (clone) in the 2TY+Km medium and the addition of IPTG as an inducer; expression at 3 h and 20 h after the addition of IPTG: 1—HBc/G-RBM; 2—HBc/G-Bep; 3—HBc/G175-CTL, 4—HBc/G161-CTL. (B) Cultivation of the same transformant in ABI-MAX auto-induction medium with glycerol and lactose; cultivation for 24 and 48 h: 1—HBc/G-RBM, 2—HBc/G-Bep, 3—HBc/G175-CTL, 4—HBc/G161-CT. M—Pierce™ Prestained Protein MW Marker (cat N 26612, Thermo Fisher Scientific Baltics UAB, Vilnius, Lithuania). Coomassie brilliant blue G-250 stained 15% SDS-PAGE gels. Figure S2. Solubility of HBc/G fusion proteins after soft lysis with a lysozyme and 0.5 M urea (SL) or disintegration via ultrasound (USD) of cells expressing recombinant proteins. Supernatant/debris distribution of target proteins after SL (A) and USD (B): Σl,—total cell lysate, (1:10); S—supernatant, (1:10); D—debris fraction after the centrifugation of the cell lysate at 13,000× *g* for 10 min; 1—HBc/G-RBM, 2—HBc/G-Bep, 3—HBc/G175-CTL, 4—HBc/G161-CTL. Coomassie brilliant blue G-250 stained 15% SDS-PAGE gels. Dilutions are shown in parenthesis. Figure S3. GF chromatography of the HBc/G175-CTL protein on a 120 mL Sepharose 4FF column with PBS, 0.5 M urea and 0.1% Tween 20. (A) Separation profile with the fraction numbers shown in red; blue line—UV, red line—conductivity. (B) SDS-PAGE analysis of the samples from the purification steps: 1—supernatant of solubilized 35% (NH4)2SO4 precipitate, (1:10); 2–9—column factions 1–8, (4:1); 10—the insoluble part (debris) after the solubilization of the precipitate, (4:1). Coomassie Brilliant Blue G-250 stained 15% SDS-PAGE gel. Dilutions are shown in parenthesis. (C) EtBr stained 1% NAGE of the samples from the purification process: 1—GeneRuler 1 kb DNA Ladder (Thermo Fisher Scientific Inc., Waltham, MA, USA), 2—VLPs of HBc/D, 3—the soluble part after the solubilization of 35% (NH_4_)_2_SO_4_ precipitate, 4–11—column factions 1–8. Figure S4. GF chromatography of the HBc/G161-CTL protein on a 120 mL Sepharose 4FF column with PBS, 0.5 M urea, and 0.1% Tween 20. (A) Separation profile of the directly loaded clarified USD lysate; (B) SDS-PAGE analysis: 1—supernatant after USD, (1:20); 2—debris after USD, (4:1); 3–10—column fractions 1–8, (4:1). Coomassie Brilliant Blue G-250 stained 15% SDS-PAGE gel. Dilutions are shown in parenthesis. (C) EtBr stained 1% NAGE: 1—GeneRuler 1 kb DNA Ladder (Thermo Fisher Scientific Inc., Waltham, MA, USA), 2–8—GF fractions 1–7. Figure S5. IEX chromatography on a Fracto-DEAE column of the HBc/G175-CTL protein. For IEX GF fractions 2–4 (Figure S3) were combined and loaded onto an IEX column with a column buffer of PBS, 0.5 M urea, and 0.1% Tween 20. (A) Separation profile: blue line—UV, violet line—conductivity; numbers of fractions are colored red. (B) SDS-PAGE analysis: 1—GF column fraction 2, (4:1) (Figure S3A), 2–9—Fracto-DEAE column fractions 3–9 (4:1), 10—GF column fraction 3, (4:1) (Figure S3A). Coomassie Brilliant Blue G-250 stained 15% SDS-PAGE. Dilutions are shown in parenthesis. (C) EtBr stained 1% NAGE: 1—GeneRuler 1 kb DNA Ladder (Thermo Fisher Scientific Inc., Waltham, MA USA), 2—GF column fraction 2 (Figure S3A), 3–9—Fracto-DEAE column fractions 3–9. Figure S6. Production of single cytokines by CD4+ and CD8+ T-cells of mice (n = 6) immunized with the HBc/G175-CTL, HBc/G161-CTL and HBc/G-Bep fusion proteins and control group of mice (n = 3) immunized with Addavax only in response to stimulation with the pooled SARS-CoV-2 CTL peptides used in this study (CTL1 + CTL2 + CTL3 + CTL4 + CTL5). Mann–Whitney U test was performed for statistical analysis; no significant differences between the groups were found. Figure S7. Production of single cytokines by CD4+ and CD8+ T-cells of mice (n = 6) immunized with the HBc/G175-CTL, HBc/G161-CTL and HBc/G-Bep fusion proteins and control group of mice (n = 3) immunized with Addavax only in response to stimulation with the unmodified recombinant HBc/G protein. GraphPad Prism was used for the presentation of the data, and the Mann–Whitney U test for the statistical analysis; the *p*-values are indicated on the top; ns = not significant. Figure S8. Production of the three cytokines by CD4+ and CD8+ T-cells of mice (n = 6) immunized with the HBc/G175-CTL, HBc/G161-CTL and HBc/G-Bep fusion proteins and control group of mice (n = 3) immunized with Addavax only in response to the stimulation with the unmodified recombinant HBc/G protein. GraphPad Prism was used for the presentation of the data, and the Mann–Whitney U test for the statistical analysis; no significant difference between the groups was found. Figure S9. (A) Neutralization of the pseudotyped SARS-CoV-2/MLV-GFP virus with the serum from mice immunized with the HBc/G-RBM protein. Each individual mouse serum (n = 6) was tested for infection inhibition at serum dilution 1:300 using HEK293/T17-ACE2-TMPRSS2 cells for infection. The neutralization effects were calculated as the proportion of GFP positive infected cells with respect to the negative control corresponded to the neutralization 0%. The data were obtained using flow cytometry. Commercially available SARS-CoV-2-neutralizing polyclonal antibodies Ab+ (Cat. No 40592-T62, Sino Biological, Beijing, China) at a dilution of 1:200 served as a positive control for neutralization. GraphPad Prism was used for the presentation of the data and statistical analysis (n = 3). (B) Correlation analysis of antibody titers (ELISA, anti-hRBM) and SARS-CoV-2/MLV-GFP virus infection neutralization for individual sera from HBc/G-RBM immunized mice. Pearson correlation coefficient and a *p*-value are indicated.
