# Supplementary material for: Toward a SARS-CoV-2 VLP Vaccine: HBc/G as a Carrier for SARS-CoV-2 Spike RBM and Nucleocapsid Protein-Derived Peptides
Source: Vaccines (Basel). 2024 Mar 4;12(3):267. doi: 10.3390/vaccines12030267 (PMC10974900; doi:10.3390/vaccines12030267)
Supplement: Supplementary file 1 [file vaccines-12-00267-s001.zip › vaccines-2815618-supplementary.pdf]

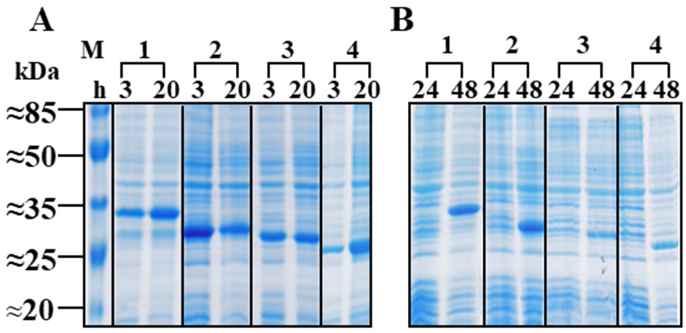

Supplementary Figure S1. Expression of the designed HBc/G fusion proteins in transformed *E. coli* BL21(DE3) cells cultivated in two different expression media. Two OD_{540} units of growing cells were pelleted, suspended in 200 μL of Laemmli buffer, incubated for 10 min at 100 °C, and 10 μL of the lysate was used for SDS-PAGE analysis. (**A**) Cultivation of a selected transformant (clone) in the 2TY+Km medium and the addition of IPTG as an inducer; expression at 3 h and 20 h after the addition of IPTG: 1—HBc/G-RBM; 2—HBc/G-Bep; 3—HBc/G175-CTL, 4—HBc/G161-CTL. (**B**) Cultivation of the same transformant in ABI-MAX auto-induction medium with glycerol and lactose; cultivation for 24 and 48 h: 1—HBc/G-RBM, 2—HBc/G-Bep, 3—HBc/G175-CTL, 4—HBc/G161-CT. M—Pierce™ Prestained Protein MW Marker (cat N 26612, Thermo Fisher Scientific Baltics UAB, Vilnius, Lithuania). Coomassie brilliant blue G-250 stained 15% SDS-PAGE gels**.**

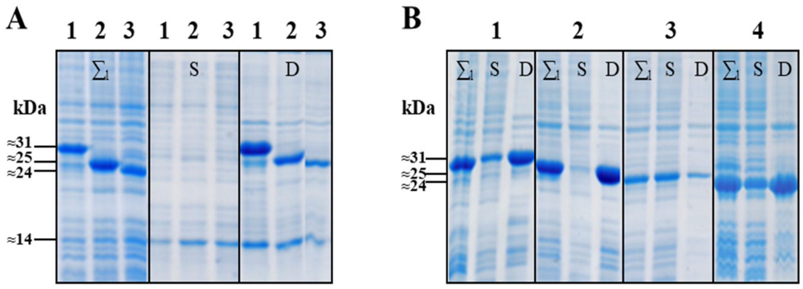

Supplementary Figure S2. Solubility of HBc/G fusion proteins after soft lysis with a lysozyme and 0.5 M urea (SL) or disintegration via ultrasound (USD) of cells expressing recombinant proteins. Supernatant/debris distribution of target proteins after SL (**A**) and USD (**B**): Σ_{l},—total cell lysate, (1:10); S—supernatant, (1:10); D—debris fraction after the centrifugation of the cell lysate at 13,000 x g for 10 min; 1—HBc/G-RBM, 2—HBc/G-Bep, 3—HBc/G175-CTL, 4—HBc/G161-CTL. Coomassie brilliant blue G-250 stained 15% SDS-PAGE gels. Dilutions are shown in parenthesis.

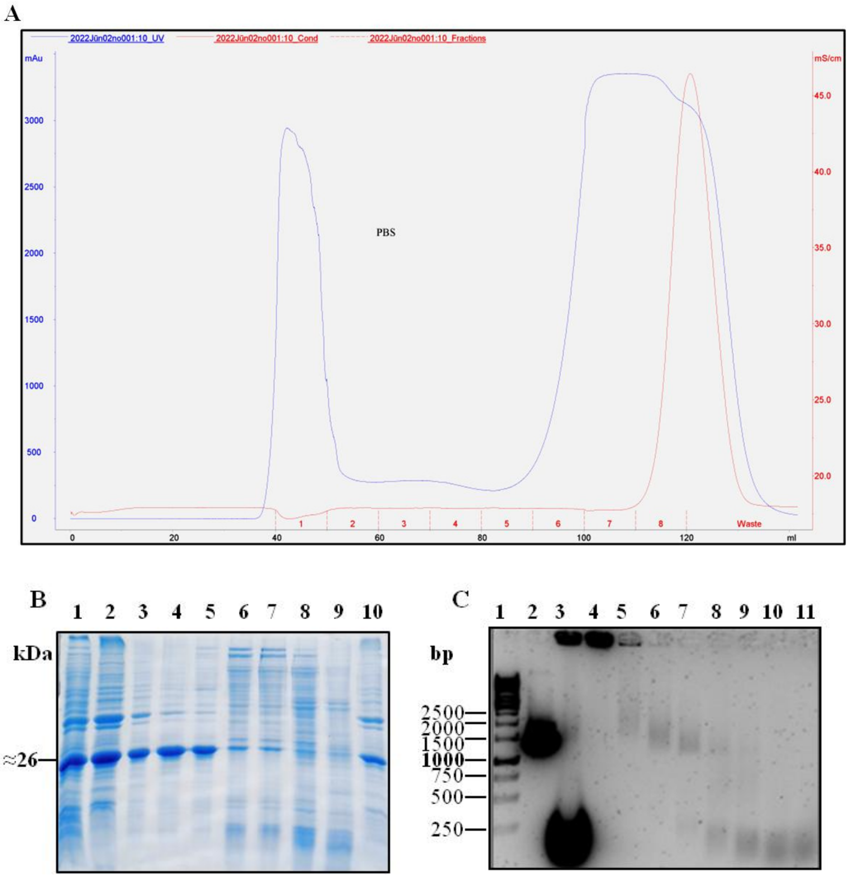

Supplementary Figure S3. GF chromatography of the HBc/G175-CTL protein on a 120 mL Sepharose 4FF column with PBS, 0.5 M urea and 0.1% Tween 20. (**A**) Separation profile with the fraction numbers shown in red; blue line—UV, red line—conductivity. (**B**) SDS-PAGE analysis of the samples from the purification steps: 1—supernatant of solubilized 35% $(NH_4)_2SO_4$ precipitate, (1:10); 2–9—column factions 1–8, (4:1); 10—the insoluble part (debris) after the solubilization of the precipitate, (4:1). Coomassie Brilliant Blue G-250 stained 15% SDS-PAGE gel. Dilutions are shown in parenthesis. (**C**) EtBr stained 1% NAGE of the samples from the purification process: 1—GeneRuler 1 kb DNA Ladder (Thermo Fisher Scientific Inc., Waltham, MA USA), 2—VLPs of HBc/D, 3—the soluble part after the solubilization of 35% $(NH_4)_2SO_4$ precipitate, 4 –11—column factions 1–8.

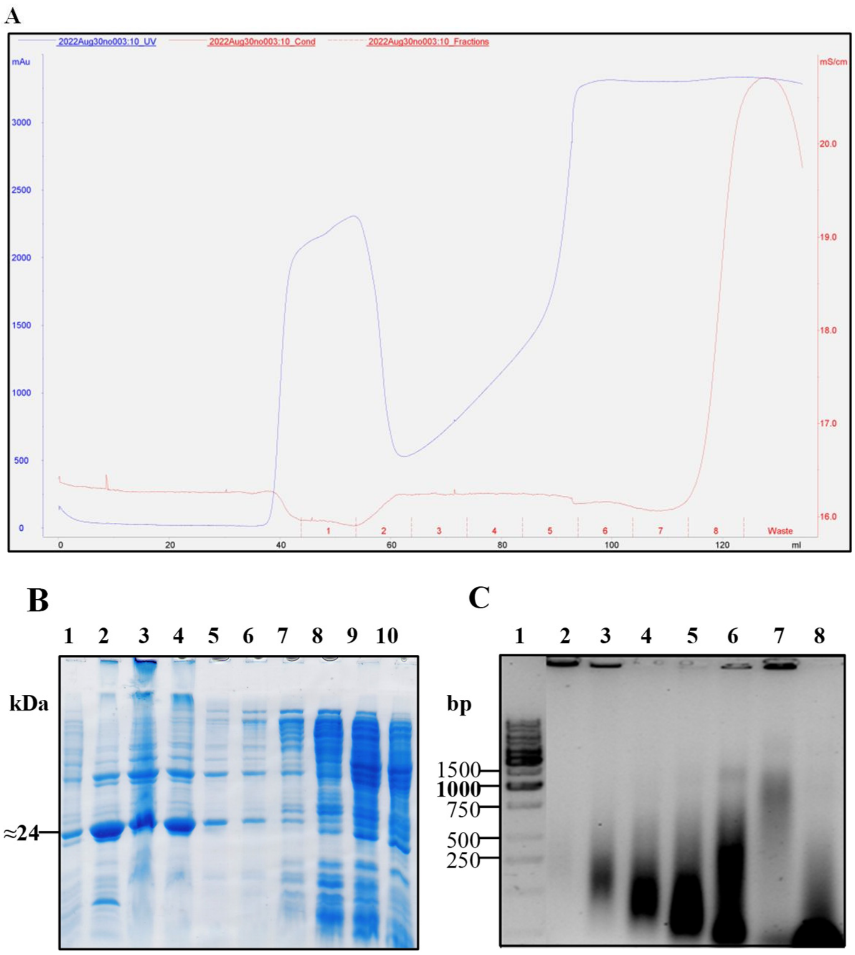

Supplementary Figure S4. GF chromatography of the HBc/G161-CTL protein on a 120 mL Sepharose 4FF column with PBS, 0.5 M urea, and 0.1% Tween 20. (**A**) Separation profile of the directly loaded clarified USD lysate; (**B**) SDS-PAGE analysis: 1—supernatant after USD, (1:20); 2—debris after USD, (4:1); 3–10—column fractions 1–8, (4:1). Coomassie Brilliant Blue G-250 stained 15% SDS-PAGE gel. Dilutions are shown in parenthesis. (**C**) EtBr stained 1% NAGE: 1—GeneRuler 1 kb DNA Ladder (Thermo Fisher Scientific Inc., Waltham, MA USA), 2–8—GF fractions 1–7.

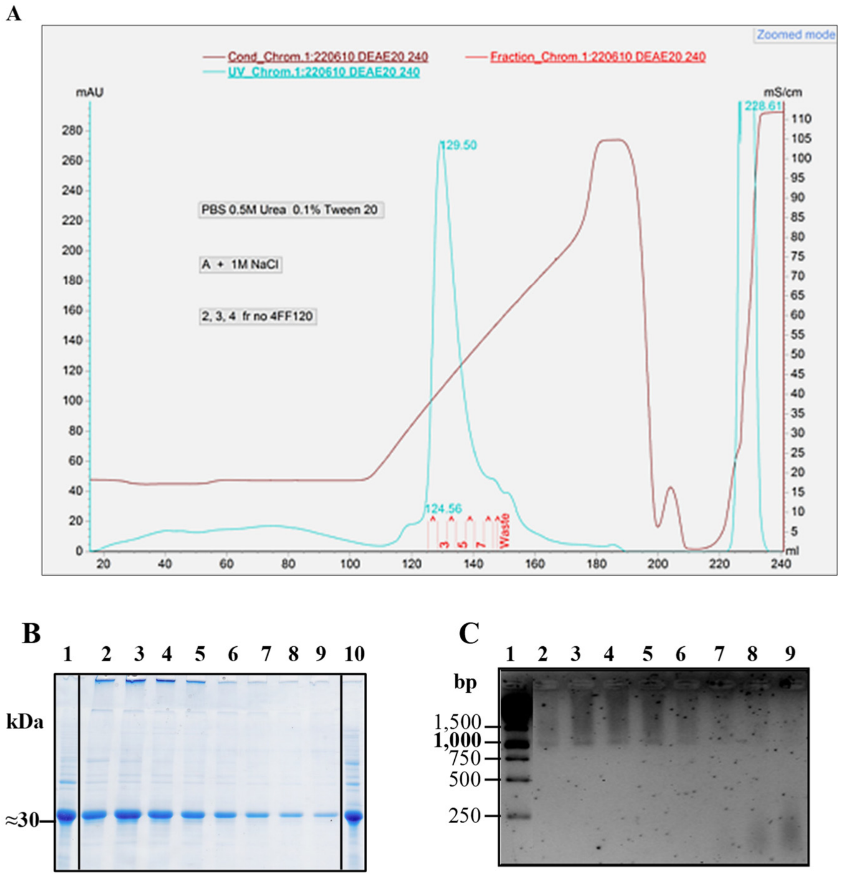

Supplementary Figure S5. IEX chromatography on a Fracto-DEAE column of the HBc/G175-CTL protein. For IEX GF fractions 2–4 (Figure S3) were combined and loaded onto an IEX column with a column buffer of PBS, 0.5 M urea, and 0.1% Tween 20. (**A**) Separation profile: blue line—UV, violet line—conductivity; numbers of fractions are colored red. (**B**) SDS-PAGE analysis: 1—GF column fraction 2, (4:1) (Figure S3**A**), 2–9—Fracto-DEAE column fractions 3–9 (4:1), 10—GF column fraction 3, (4:1) (Figure S3**A**). Coomassie Brilliant Blue G-250 stained 15% SDS-PAGE. Dilutions are shown in parenthesis. (**C**) EtBr stained 1% NAGE: 1—GeneRuler 1 kb DNA Ladder (Thermo Fisher Scientific Inc., Waltham, MA USA), 2—GF column fraction 2 (Figure S3**A**), 3–9—Fracto-DEAE column fractions 3–9

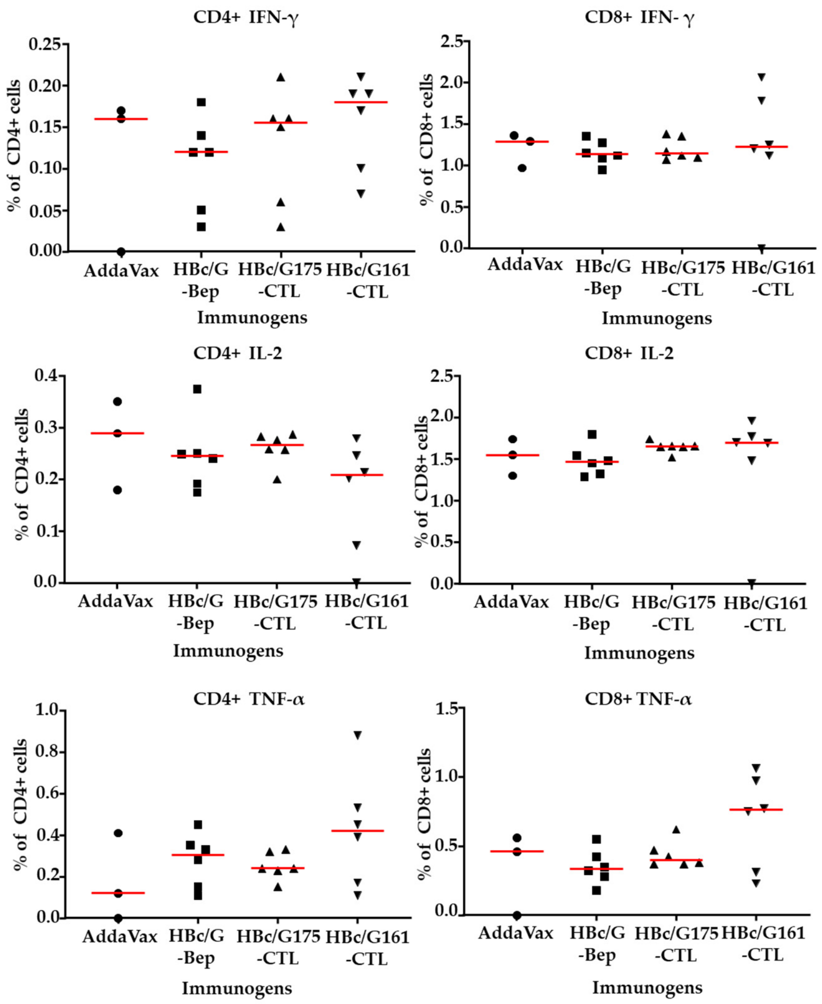

Supplementary Figure S6. Production of single cytokines by CD4+ and CD8+ T-cells of mice (n=6) immunized with the HBc/G175-CTL, HBc/G161-CTL and HBc/G-Bep fusion proteins and control group of mice (n=3) immunized with Addavax only in response to stimulation with the pooled SARS-CoV-2 CTL peptides used in this study (CTL1+CTL2+CTL3+CTL4+ CTL5). Mann–Whitney U test was performed for statistical analysis; no significant differences between the groups were found.

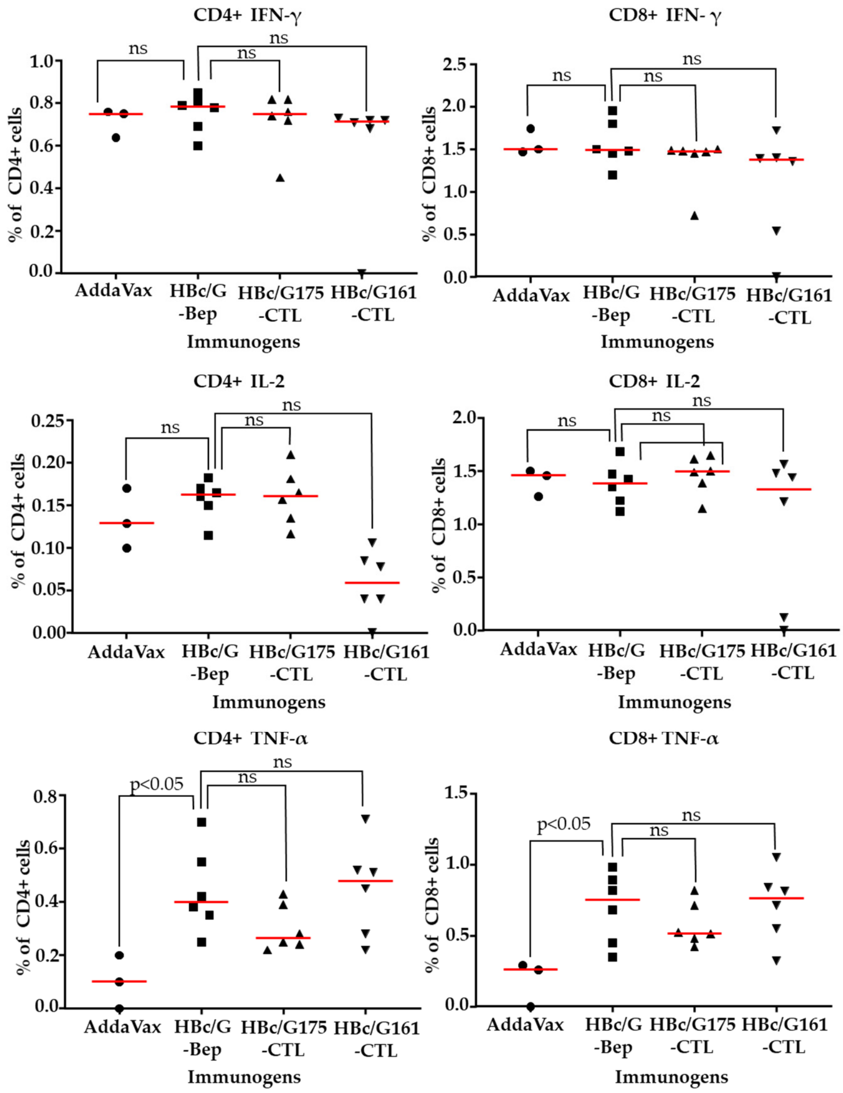

Supplementary Figure S7. Production of single cytokines by CD4+ and CD8+ T-cells of mice (n=6) immunized with the HBc/G175-CTL, HBc/G161-CTL and HBc/G-Bep fusion proteins and control group of mice (n=3) immunized with Addavax only in response to stimulation with the unmodified recombinant HBc/G protein. GraphPad Prism was used for the presentation of the data, and the Mann–Whitney U test for the statistical analysis; the p-values are indicated on the top; ns = not significant.

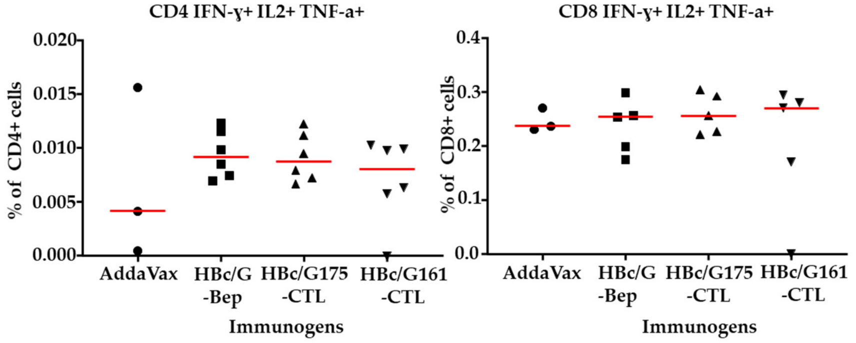

Supplementary Figure S8. Production of the three cytokines by CD4+ and CD8+ T-cells of mice (n=6) immunized with the HBc/G175-CTL, HBc/G161-CTL and HBc/G-Bep fusion proteins and control group of mice (n=3) immunized with Addavax only in response to the stimulation with the unmodified recombinant HBc/G protein. GraphPad Prism was used for the presentation of the data, and the Mann–Whitney U test for the statistical analysis; no significant difference between the groups was found.

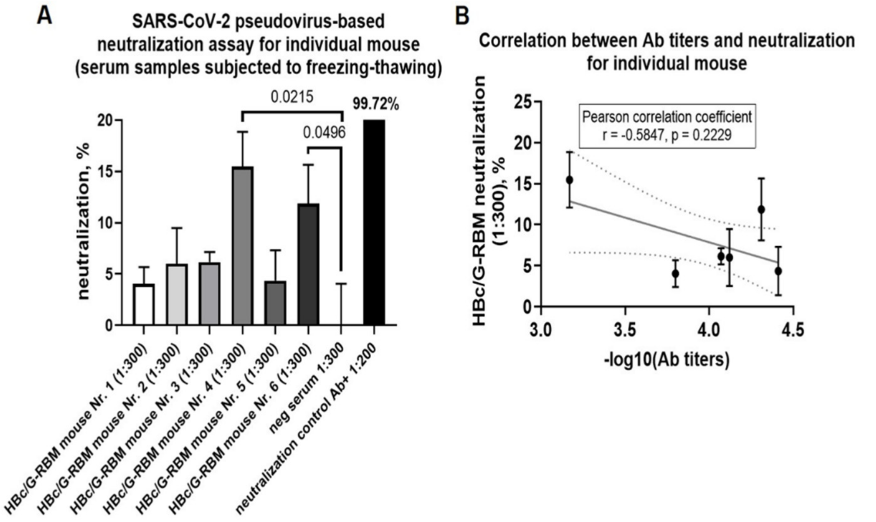

Supplementary Figure S9. (**A**) Neutralization of the pseudotyped SARS-CoV-2/MLV-GFP virus with the serum from mice immunized with the HBc/G-RBM protein. Each individual mouse serum (n=6) was tested for infection inhibition at serum dilution 1:300 using HEK293/T17-ACE2-TMPRSS2 cells for infection. The neutralization effects were calculated as the proportion of GFP positive infected cells with respect to the negative control corresponded to the neutralization 0 %. The data were obtained using flow cytometry. Commercially available SARS-CoV-2-neutralizing polyclonal antibodies Ab+ (Cat. No 40592-T62, Sino Biological, Beijing, P.R.China) at a dilution of 1:200 served as a positive control for neutralization. GraphPad Prism was used for the presentation of the data and statistical analysis (n=3). (**B**) Correlation analysis of antibody titers (ELISA, anti-hRBD) and SARS-CoV-2/MLV-GFP virus infection neutralization for individual sera from HBc/G-RBM immunized mice. Pearson correlation coefficient and a p values are indicated.
